# Correcting for Sequencing Error in Maximum Likelihood Phylogeny Inference

**DOI:** 10.1534/g3.114.014365

**Published:** 2014-11-04

**Authors:** Mary K. Kuhner, James McGill

**Affiliations:** Department of Genome Sciences, University of Washington, Seattle, Washington 98195-5065

**Keywords:** sequencing error, phylogeny inference, maximum likelihood

## Abstract

Accurate phylogenies are critical to taxonomy as well as studies of speciation processes and other evolutionary patterns. Accurate branch lengths in phylogenies are critical for dating and rate measurements. Such accuracy may be jeopardized by unacknowledged sequencing error. We use simulated data to test a correction for DNA sequencing error in maximum likelihood phylogeny inference. Over a wide range of data polymorphism and true error rate, we found that correcting for sequencing error improves recovery of the branch lengths, even if the assumed error rate is up to twice the true error rate. Low error rates have little effect on recovery of the topology. When error is high, correction improves topological inference; however, when error is extremely high, using an assumed error rate greater than the true error rate leads to poor recovery of both topology and branch lengths. The error correction approach tested here was proposed in 2004 but has not been widely used, perhaps because researchers do not want to commit to an estimate of the error rate. This study shows that correction with an approximate error rate is generally preferable to ignoring the issue.

As originally developed, maximum likelihood (ML) phylogeny inference assumes that the data are known without error (Felsenstein 1981). A straightforward extension to incorporate a known sequencing error rate was proposed by Felsenstein (2004). As far as we know it has never been implemented despite its obvious potential usefulness. We speculate that the correction has been neglected because it requires committing to an estimate of the error rate, and researchers fear that an inaccurate correction would be worse than no correction at all.

We have implemented sequencing error correction in the PHYLIP (Felsenstein 2005) programs Dnaml and Dnamlk, which infer phylogenies via DNA-based ML with a molecular clock (Dnamlk) or without one (Dnaml). Using simulated data, we test both the usefulness of error correction in obtaining a correct phylogeny and its vulnerability to misstatement of the sequencing error rate.

Topological accuracy of phylogenies is important for purposes such as taxonomic classification (see, for example, the discussion in De Queiroz and Gauthier 1990) and detection of cospeciation patterns (for example, Machado *et al.* 2005). Accuracy of branch lengths is critical in any use of phylogenies in the context of time, such as dating of key events (reviewed in Rutschmann 2006), inference of mutation rates (for example, Drummond *et al.* 2006), and parameter inference via coalescent theory (for example, Kuhner 2009). Thus, sequencing error correction is broadly relevant to the use of ML phylogenetics. We also discuss its potential use in Bayesian phylogenetics.

## Materials and Methods

### Sequencing error correction

In the standard DNA- or RNA-based ML algorithm, values stored at the tips of the tree indicate the probability of the observed data given the underlying true base. For example, in a no-error case when base A is observed, the four values stored will be (1,0,0,0), corresponding to a probability of 1 for (A observed|true base A) and a probability of 0 for (A observed|true base C, G, or T/U). These values form the basis for the peeling algorithm (Felsenstein 1981), which calculates probabilities working from the tips back to the root. Note that these numbers represent the probability of the observation, not the probability of the underlying base, and need not sum to 1; for example, a completely uninformative observation (missing data) corresponds to values of (1,1,1,1).

Following Felsenstein (2004), we use a simple model of sequencing error in which a base is misread as a random different base with probability *ε* and this probability is the same across sequences and sites. Under this model, the probability of observing A given that the underlying base is A becomes 1 − *ε*, and the probability of observing each of C, G, or T, given that the underlying base is A, becomes *ε*/3. More complex models, including sequence-specific or site-specific error rates, can readily be derived by the same approach. (This discussion assumes DNA, but RNA is handled identically, substituting U for T.)

Nucleotide ML algorithms often allow for the International Union of Pure and Applied Chemistry nucleotide ambiguity codes (Cornish-Bowden 1985). To handle these, we must decide what an observation of, say, M (meaning that the base is ambiguous between A and C) implies. We assume that sequencing error generates incorrect calls of specific bases, which are then converted to ambiguity codes. (This relieves us from needing a separate model for the probability of observing M if the underlying base is A.) Under this assumption, if the true base is A, an observation of M could result either from a correct call of A (probability 1 − *ε*) or an erroneous call of C (probability *ε*/3) and therefore has probability 1 − 2*ε*/3, and similarly if the true base is C. If the true base is G, the chance of observing M is the chance of an erroneous call of G as either A or C, or 2*ε*/3, and similarly for a true base T. Similar reasoning gives tip values for the other IUPAC codes. A representative sample of these probabilities is given in Table 1.

**Table 1:** Example probabilities for resolved nucleotides and IUPAC ambiguity codes under an error model

Code	Meaning	Probabilities (A, C, G, T)
A	A	1 − *ε*, *ε*/3, *ε*/3, *ε*/3
M	A or C	1 − 2*ε*/3, 1 − 2*ε*/3, 2*ε*/3, 2*ε*/3
V	A or C or G	1 − *ε*/3, 1 − *ε*/3, 1 − *ε*/3, *ε*
N	Any base	1, 1, 1, 1

IUPAC, International Union of Pure and Applied Chemistry**.**

An analogous approach could be used for amino acid ML algorithms. Application to codon-based algorithms would be more complex but is possible in principle.

### Simulation design

We used the program *rantree.c* (J. Felsenstein, unpublished data) to create random clocklike branching-process trees of 20 tips for a given value of the tree-size scaling parameter *t*. The *t* parameter establishes the scaling of the tree: for example, the interval between the rootward and next-rootward splits has an expected mean length of *t*/2. We then used the program *rectreedna.c* (J. Felsenstein and M. Kuhner, unpublished data) to simulate 20,000 bp of DNA data per taxon on these trees using the Kimura 2-parameter model with transition/transversion rate 2.0. We inferred trees from these DNA data with versions of Dnaml and Dnamlk from PHYLIP 3.69 (Felsenstein 2005), which was augmented with the error correction described previously. For each value of *t*, 100 trees were simulated, and a no-error data set was made for each tree; with-error data sets were derived by independent addition of varying degrees of error to these 100 data sets.

We considered a range of true values of *ε* and *t*, as shown in Table 2, which also gives mean single-nucleotide polymorphisms (SNPs) per kilobase. The proportion of SNPs due to error rather than mutation can be roughly inferred by comparing the zero-error case to the others. We then inferred trees using no error correction and a range of error corrections bracketing the true value, or in cases where the true error was zero, from 0 to 10^−2^. We did not simulate the most unbalanced cases: for these sequence lengths, when *e* = *t* ∗ 1000 inference is expected to be nearly impossible, and when *e* = *t*/1000 error will have almost no effect.

**Table 2:** Mean SNPs per kilobase in data sets for each condition

Error	Scaling Parameter *t*		
	10^−4^	10^−3^	10^−2^
0.0	4.8	47.2	376.4
10^−4^	6.75	49.1	ND
10^−3^	24.4	65.9	388.8
10^−2^	185.8	220.7	489.8
10^−1^	ND	883.9	923.5

SNPs, single-nucleotide polymorphism; ND, not done.

To compare the inferred trees to the true trees, we used the topology-only metric of Robinson and Foulds (“RF,” Robinson and Foulds 1981) to assess topological correctness, and the branch-length metric of Robinson and Foulds (“RFL,” Robinson and Foulds 1979) to assess correct recovery of branch lengths. Our simulation conditions were chosen to represent between-species phylogenies (hence the use of branching-process rather than coalescent trees) and to explore both fairly easy and more difficult phylogeny reconstructions. We used both RF and RFL because of the finding (M. K. Kuhner and J. Yamato 2014) that branch-length tree comparison metrics are more informative for closely related trees (corresponding in this case to highly successful inferences) and topology-only metrics are more informative for discordant trees (relatively poor inferences).

## Results

We show our main results as a series of figures relating actual and declared error to phylogenetic inference accuracy. In the upper panels, RF measures accuracy of the inferred topology only; an increase of 2 units indicates an average of 1 additional error per inferred tree. In the lower panels, RFL measures inference of topology and branch length; its values should be considered relative to the tree scaling factor *t*. (We did not rescale proportionate to *t* as in some plots this obscures readability.) Throughout, we observe that trees with *t* = 10^−4^ (red lines) had poor topological accuracy due to a scarcity of SNPs, whereas trees with greater values of *t* were well inferred topologically (and had relatively more accurate branch lengths) unless error intervened.

For comparison purposes, Sanger sequencing can achieve error rates down to 10^−4^ (Ewing and Green 1998), while estimated error rates for next-generation sequencing methods vary between 10^−3^ and 4 × 10^−2^ (Glenn 2014). Error rates can be further reduced from these levels by taking a consensus of multiple reads. Even in sequences with high read depth, errors can be introduced in assembly: the frequency of such errors depends on genome, algorithm, and the error rate of the underlying sequencing method (Haiminen *et al.* 2011).

**Figure 1:**
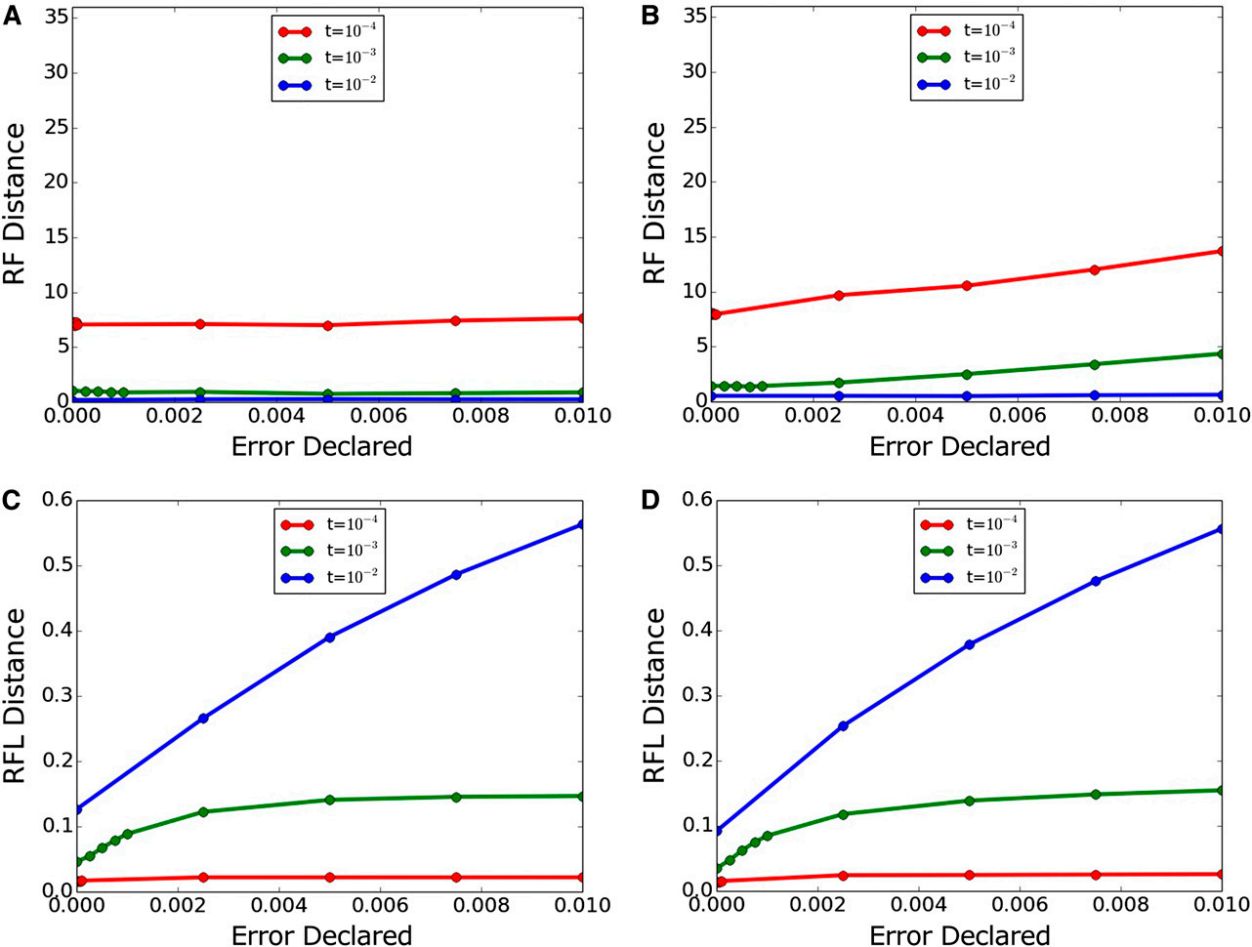
Inference accuracy with true error 0. (A, C) Dnaml and (B, D) Dnamlk. (A, B) RF (topology only) and (C, D) RFL (topology and branch length). RF, topology-only metric of Robinson and Foulds; RFL, branch-length metric of Robinson and Foulds.

Figure 1 shows results when an error correction is erroneously imposed on data generated without error. Dnaml’s topology inference is robust across the range of declared error tested here, whereas Dnamlk sees some worsening of topological inference with high declared error values. Both algorithms see worsening of branch-length inference with increased declared error; this was least pronounced with the highly informative *t* = 10^−2^ trees.

**Figure 2:**
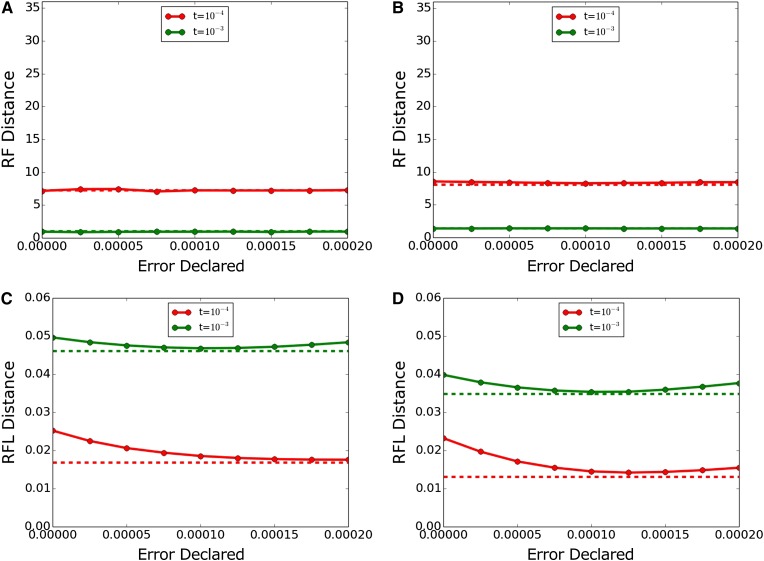
Inference accuracy with true error 10^−4^. (A, C) Dnaml and (B, D) Dnamlk. (A, B) show RF (topology only) and (C, D) show RFL (topology and branch length). Dashed lines indicate performance with actual error 0 and declared error 0 for comparison. RF, topology-only metric of Robinson and Foulds; RFL, branch-length metric of Robinson and Foulds.

Figure 2 shows results with a true error of 10^−4^ miscalls per base. Topological accuracy was unaffected by error correction within the bounds tested here. Branch length inference was improved by error correction, particularly for the lower value of *t*. The apparent optimal value of the correction was somewhat higher than the actual error rate, and there was only modest worsening with overcorrection up to 2x the actual error rate. Both algorithms recovered branch lengths very close to the no-error case (dashed lines) using their optimal correction values. Dnamlk recovered branch lengths more successfully than Dnaml, as expected for data which match Dnamlk’s clock assumption.

**Figure 3:**
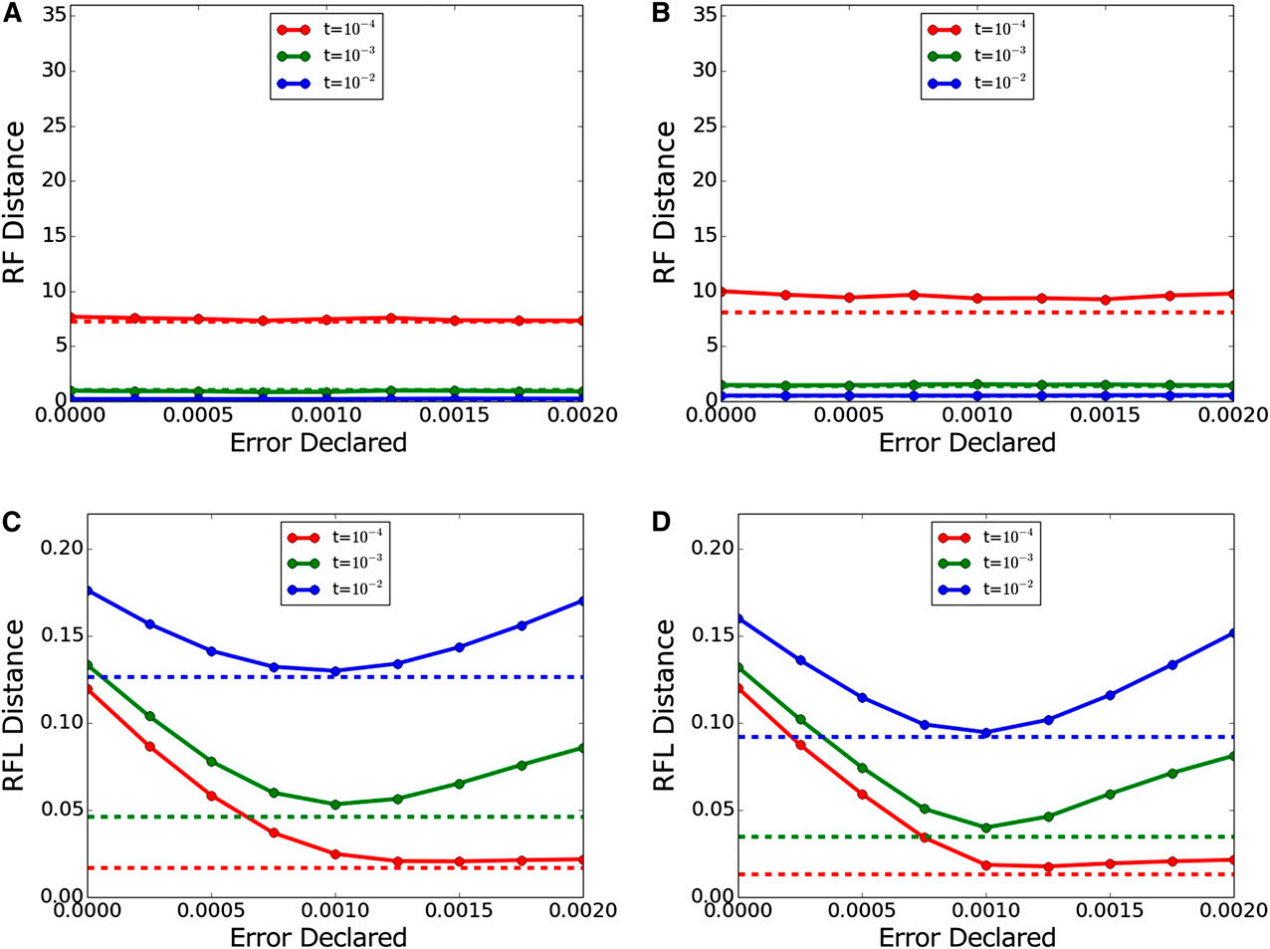
Inference accuracy with true error 10^−3^. (A, C) Dnaml and (B, D) Dnamlk. (A, B) RF (topology only) and (C, D) RFL (topology and branch length). Dashed lines indicate performance with actual error 0 and declared error 0 for comparison. RF, topology-only metric of Robinson and Foulds; RFL, branch-length metric of Robinson and Foulds.

Figure 3 shows results with a true error of 10^−3^ miscalls per base. Again, topological accuracy was unaffected, but branch length inference improved markedly with correction, especially for *t* = 10^−4^. Although branch lengths worsened with overcorrection in most cases, this was generally less severe than noncorrection.

**Figure 4:**
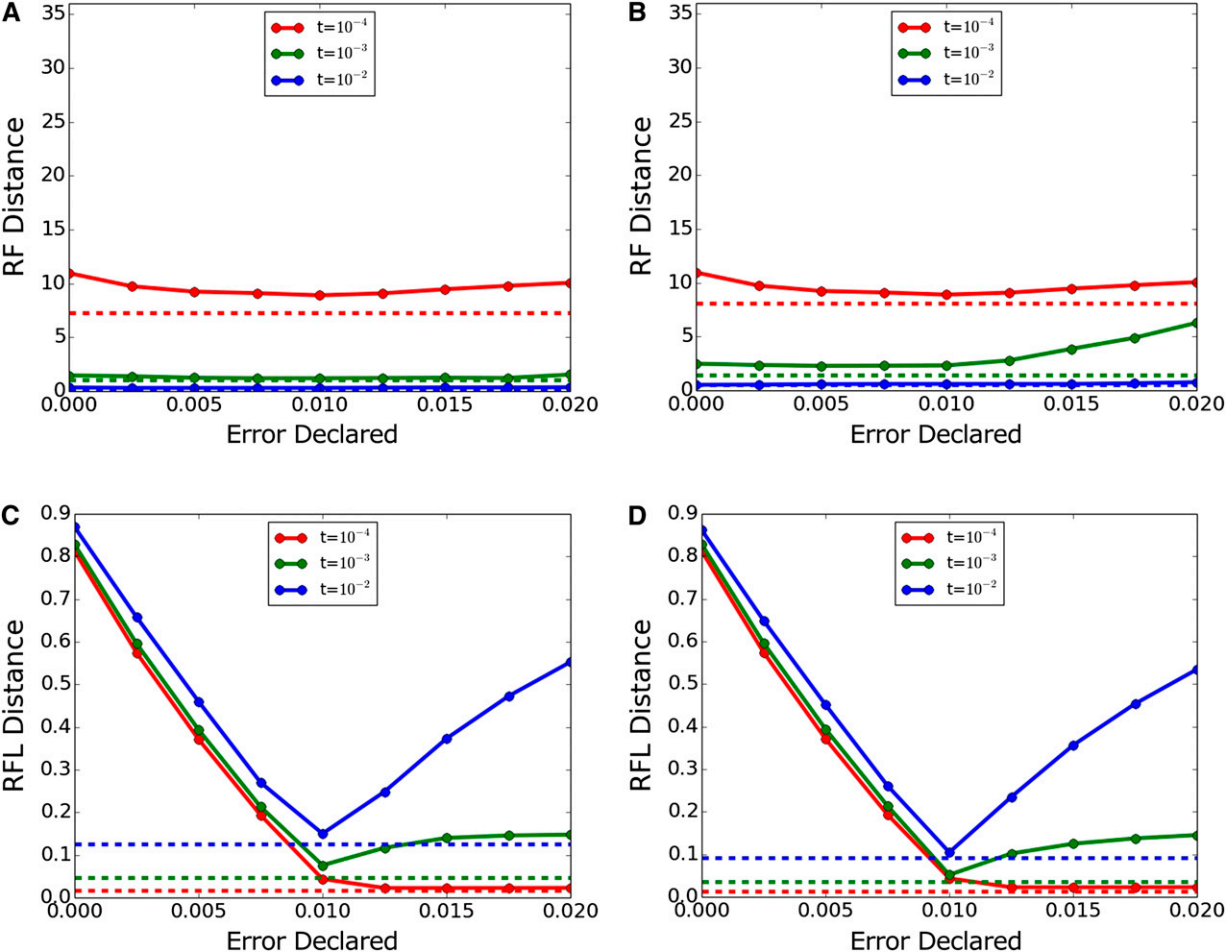
Inference accuracy with true error 10^−2^. (A, C) Dnaml and (B, D) Dnamlk. (A, B) RF (topology only) and (C, D) RFL (topology and branch length). Dashed lines indicate performance with actual error 0 and declared error 0 for comparison. RF, topology-only metric of Robinson and Foulds; RFL, branch-length metric of Robinson and Foulds.

At a still higher error rate of 10^−2^ miscalls per base, Figure 4 shows topological inference beginning to react negatively to overcorrection, especially in Dnamlk and with lower values of *t*. At this error rate, the effect of the correction on branch length inference is dramatic, with undercorrection worse than overcorrection in all cases. A novel pattern appears for *t* = 10^−4^ (red line) in which branch length inference apparently continues to improve beyond the point where declared error equals true error.

**Figure 5:**
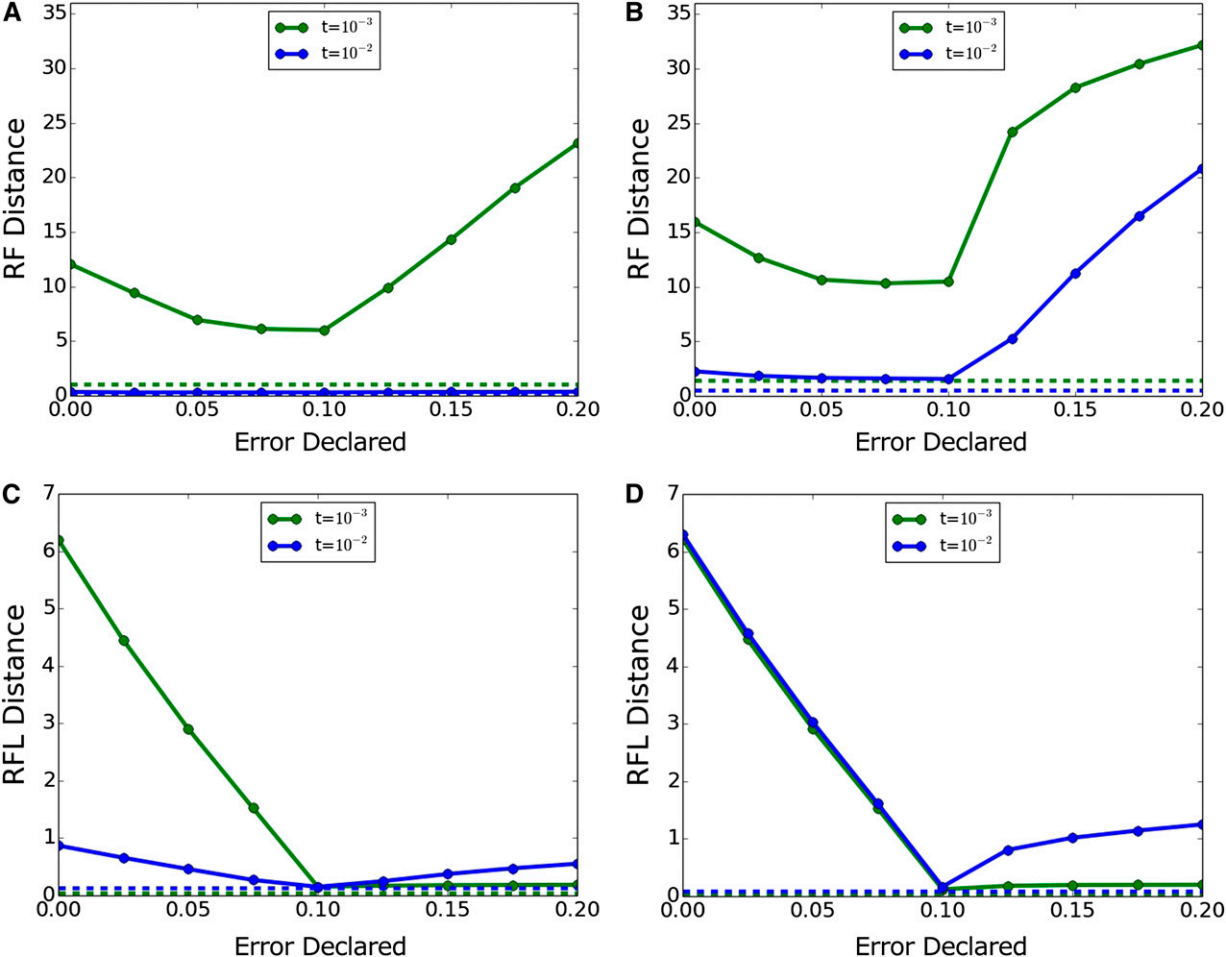
Inference accuracy with true error 10^−1^. (A, C) Dnaml and (B, D) Dnamlk. (A, B) RF (topology only) and (C, D) RFL (topology and branch length). Dashed lines indicate performance with actual error 0 and declared error 0 for comparison. RF, topology-only metric of Robinson and Foulds; RFL, branch-length metric of Robinson and Foulds.

Finally, at the enormous error rate of 10^−1^ miscalls per base, Figure 5 shows this tendency much more strongly. Topological inference worsens with both undercorrection and, especially, overcorrection. Branch length inference is very poor with undercorrection, decreases to a level close to the no-error case with correction, and then increases only slightly with overcorrection; but this apparently correct branch length inference is associated with increasingly poor topology inference.

**Figure 6:**
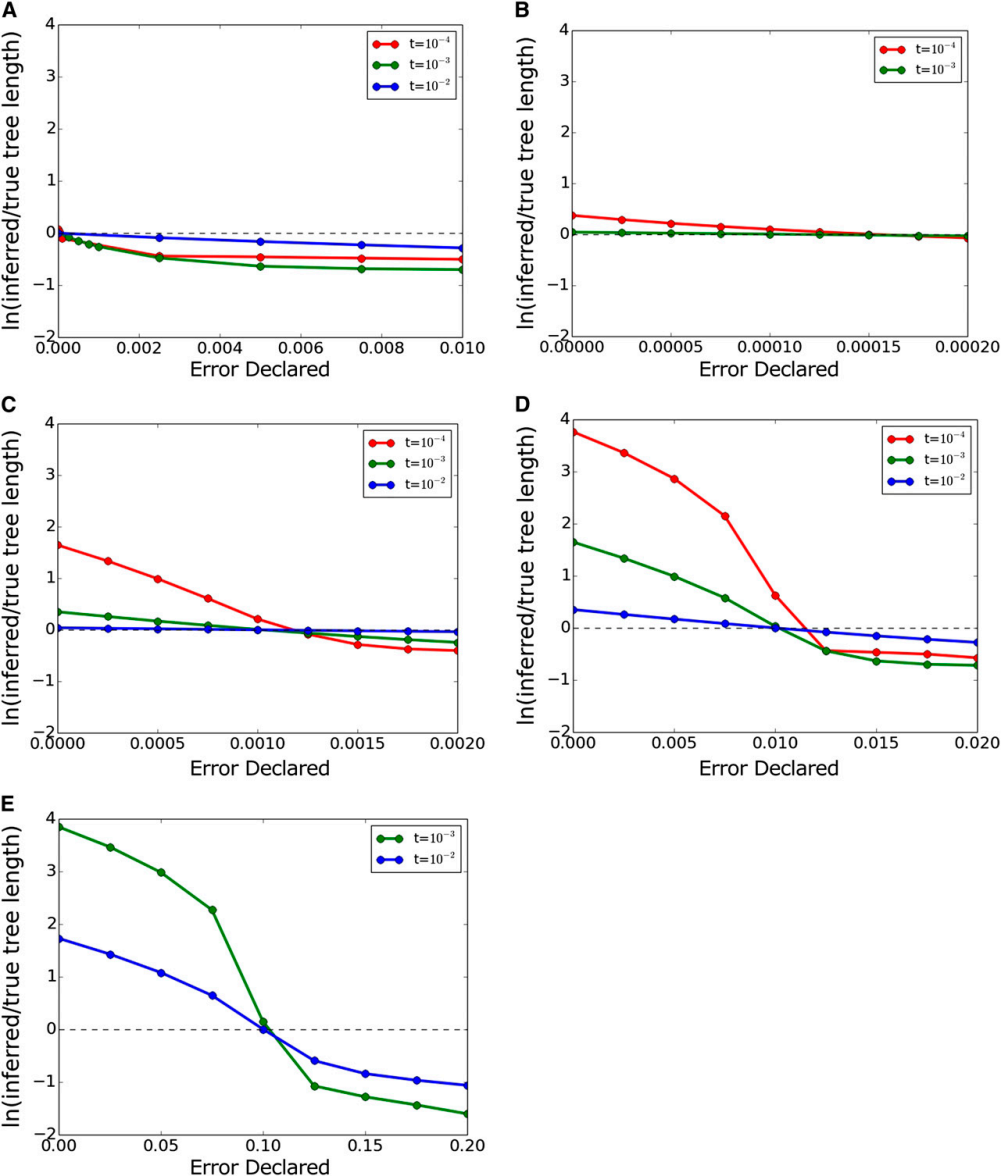
Ratio of inferred tree length to true tree length as a function of declared error. Dashed line indicates equality of inferred and true length. (A) True error 0.0. (B) True error 10^−4^. (C) True error 10^−3^. (D) True error 10^−2^. (E) True error 10^−1^.

We hypothesized that this pattern of apparently good branch length inference combined with increasingly poor topology inference represents inferred trees with extremely short branches and semi-randomized topologies due to interpretation of essentially all of the data as sequencing error. To test this, we plotted the mean length of the Dnaml inferred trees divided by the mean length of the trees on which the data were generated. These plots are shown in Figure 6, with the tree length ratio plotted on a natural-log scale. Figure 6 makes it clear that branch lengths are fairly robust to error correction except when the true error is greater than *t*. For extreme error cases, however, mean tree length starts out much too high in the inferred trees, becomes correct around the point where declared error is equal to true error, and then becomes much too low with further correction. This confirms our understanding of the anomalous results in Figures 4 and 5: branch lengths appear good only because they are extremely short, and topology cannot be inferred because the ordering of these short branches is randomized.

## Discussion

### Error correction generally improves inference

In almost all of the cases examined, declaration of the true error rate produced the most accurate branch lengths; in a few cases with very low error, slight overcorrection was actually superior. Topology inference was less sensitive to error, but in cases in which it varied with error rate, declaration of the true error rate again produced the best results.

Some improvement was seen even when the error rate was quite low (see Figure 2), showing that the correction is not deadweight even for highly accurate sequences. However, the greater the error rate the greater the need for the correction. Somewhat to our surprise, even with an error rate of 0.01 errors per base, recovery of branch lengths with a correct declared error was only a little worse than the associated no-error case.

### Aggressive overcorrection can lead to pathologies

A declared error of 0.2 errors/base, applied to data with an actual error of 0.1 errors/base and a low tree scaling (so that nonerror SNPs were rare), caused essentially all of the variable sites to be interpreted as error, resulting in extremely short branch lengths and randomized topologies. In general, overcorrection appeared problematic when *ε* exceeded *t*, but we did not test intermediate values of *ε* so this cannot be treated as an exact rule.

### Type of inference matters

When error was low, the clock-assuming program Dnamlk produced more accurate branch lengths than Dnaml, presumably because it has fewer degrees of freedom in branch length inference. However, as error increased Dnamlk showed increasing difficulties. The error correction implementation is identical in both programs, but in Figure 5 Dnamlk performed substantially worse than Dnaml. This is particularly striking in that these data were simulated with a molecular clock. The clock constraint means that sufficient distortion of branch lengths can lead to an incorrect topology. For phylogenetic inference in the context of very dirty data, non-clocklike methods are more robust and should be preferred unless the clock assumption is essential (for example, in phylogenetic dating).

The results presented here involve ML inference. Bayesian inference with a reasonable prior on *ε* might be able to tolerate higher declared error rates than ML for two reasons: the prior could help to direct attention away from zero-length trees, and even if near-zero trees were produced they would be accompanied by longer trees and the credibility intervals would therefore reflect topological signal. Presenting the single ML tree, in contrast, foregrounds the “all SNPs are errors” solution even though more resolved trees would have only slightly lower likelihood. This error correction should be tested in Bayesian inference programs with *ε* as a parameter. It would also be interesting to see whether the relaxed clock approach of Drummond *et al.* (2006) could be combined with sequencing error correction to allow successful inference of clocklike trees in the presence of extreme sequencing error.

### Sequencing error may not be constant

We have assumed that all sequences in a data set have the same error rate. This will not always be true in practice due to variation in sequencing methods, read depth, sample condition, and other factors. If the error rates of various sequences are known, it is trivial to set a separate *ε* for each one: we plan to add this capability to upcoming versions of our software. When the rates are unknown, any constant value of *ε* will be wrong for some sequences. Our results suggest that tree distortion is likely if the unknown values vary by more than a factor of two. Novel analytic tools will be needed to correct the inference in such cases. In the meantime, it is likely that even use of an average correction which is wrong for some sequences will be better than use of no correction, which is wrong for all sequences.

We have also assumed that errors occur independently across the length of the sequence. This is not necessarily true: for example, assembly-based errors may tend to occur in clusters where the wrong fragment has been chosen. For conventional phylogenetic applications like the ones studied here, the main effect of this clustering is to introduce additional stochastic variability into the error rate per sequence, and the comments above apply. Clustered errors are a greater concern for models in which site locations are significant: codon likelihood models, models of secondary structure, and ancestral recombination graphs. Further work will be needed to appropriately handle clustered sequencing error in such models.

We find that the sequencing error correction for ML analysis proposed by Felsenstein (2004) improves recovery of branch lengths across a wide range of parameter values. In general, overcorrection does less harm than undercorrection. For any but the most extreme sequencing error rates, there is little effect of error correction on topological inference. When the error rate is extremely high, however, values of the declared error different from the true error are poorly tolerated in both branch length inference and topology inference, with undercorrection particularly detrimental to branch length inference and overcorrection to topological inference.

We strongly recommend incorporation of sequencing error correction into ML phylogeny algorithms. In human SNP typing, it has been common to rely on allele frequency cutoffs as a correction for sequencing error (since independent errors generally manifest as singleton SNPs), but McGill *et al.* (2013) have shown that the sequencing error correction used here is more statistically powerful than omission of rare SNPs, which also removes considerable signal from the data. While this study did not test the usefulness of specifying sequence-specific error rates, it is very likely that a best-practices implementation should include this capability.

It will also be important for the creators of sequencing and assembly pipelines to develop and publish accurate estimates of their error rates, in order to facilitate correct analysis of the resulting data. Although inference of the sequencing error rate from phylogenetic data might be possible, direct measurement will be more powerful and should be prioritized. When a study contains sequences of very different quality (ancient *vs.* modern DNA, model *vs.* nonmodel organisms, shallow *vs.* deep read depth, different sequencing platform) it will be important to develop and use sequence-specific error rates.

## Software Availability

The sequencing error correction described here has been implemented in the LAMARC program since version 2.1.5. It will also be implemented in the next release of PHYLIP. The unpublished programs *rantree.c* and *rectreedna.c* used to simulate and analyze data are archived in Supplementary Materials File S1, along with example parameter files.

## Supplementary Material

Supporting Information
