# Supplementary material for: Correcting for Sequencing Error in Maximum Likelihood Phylogeny Inference
Source: G3 (Bethesda). 2014 Nov 4;4(12):2545–52. doi: 10.1534/g3.114.014365 (PMC4267948; doi:10.1534/g3.114.014365)
Supplement: Supporting Information [file supp_4_12_2545__index.html]

Correcting for Sequencing Error in Maximum Likelihood Phylogeny Inference — Supporting Information 

# Correcting for Sequencing Error in Maximum Likelihood Phylogeny Inference

## Supporting Information for Kuhner and McGill, 2014

**Files in this Data Supplement:**

- File S1 - Contains the unpublished programs rantree.c and rectreedna.c, which were used to simulate and analyze the data. (.tar, 30 KB)
